# Evidence for the Existence of Two Opposing Pulse Waves in Retinal Vein Segments Within the Optic Disc

**DOI:** 10.1167/iovs.66.15.43

**Published:** 2025-12-15

**Authors:** Aleksandar Vukmirovic, William H. Morgan, Danail Obreschkow, Anmar Abdul-Rahman, Dao-Yi Yu, Andrew Mehnert

**Affiliations:** 1 Lions Eye Institute, Centre for Ophthalmology and Visual Science, University of Western Australia, Perth, Western Australia, Australia; 2 International Space Centre, Crawley, Western Australia, Australia; 3 International Centre for Radio Astronomy Research, University of Western Australia, Crawley, Western Australia, Australia; 4 Department of Ophthalmology, Counties Manukau District Health Board, Auckland, New Zealand

**Keywords:** Pulse wave velocity, retinal venous pulsation, optic disc, modified photoplethysmography, phase

## Abstract

**Purpose:**

Pulse wave velocity (PWV) is used to assess vascular disease. We recently introduced a non-invasive technique for measuring retinal PWVs and presented evidence of venous pulse wave propagation opposite blood flow in the central optic disc close to maximum pulsation. The purpose of this study was to characterize venous pulse wave propagation more peripherally in the optic disc, where little is currently known about its dynamics.

**Methods:**

We measured PWVs in optic disc veins in six healthy eyes at varying intraocular pressures (57 measurements in total). Our technique involves acquiring video of the retina over three cardiac cycles, fitting a harmonic regression model to the wave-like pattern of green channel intensities at each point along the vessel centerline, and using the differences in wave timing (phase) to calculate PWVs.

**Results:**

The mean age was 42.3 ± 18.6 years, and the mean intraocular pressure was 33.2 ± 13.3 mm Hg. All distance–phase plots showed a linear down trend followed by a linear up trend, suggesting a PWV retrograde to blood flow before the turning point and anterograde thereafter. The turning point was located a mean distance of 0.14 ± 0.12 mm from the point of maximum pulse amplitude. The distances of the turning point and maximum pulse amplitude from the optic disc center were positively associated (*P* = 0.013).

**Conclusions:**

These results suggest that two opposing pulse waves travel along retinal vein segments within the optic disc, raising the possibility that at least two distinct pulse wave generators exist either side of the lamina cribrosa.

A wave is a disturbance that travels through a medium in which energy continuously shifts between kinetic and potential forms.^1^ The vessel pulse wave is a mechanical wave that propagates along the vessel walls driven by pulsatile pressure fluctuations during the cardiac cycle.^1^^,^^2^ Pulse wave velocity (PWV) is the speed and direction of travel of this wave. Arterial PWVs can be measured using a variety of techniques.^3^ They have been used clinically to determine vessel stiffness in order to assess vascular health and manage cardiovascular disease.^4^ Venous PWVs are generally not measured because the pulse signal is typically much weaker. However, we recently developed a method for measuring venous PWVs based on measuring the phase shift of the pulse wave at multiple sites along a retinal vein within the optic disc.^2^ This ability to measure venous PWVs may be useful given the commonality of venous occlusive disease systemically and in the eye.^5^

Physical modeling experiments, using fluid flow within collapsible tubing inside pressurized chambers, have shown that downstream flow resistance reduces pulsation amplitude.^6^ Indeed, we have observed this in patients with retinal venous occlusive disease, where they had reduced venous pulse amplitudes.^7^ We have previously proposed a model to explain this phenomenon wherein pulse wave transmission is reduced across the downstream resistance element, which also elevates upstream intramural venous pressure, reducing vessel compliance with a combined effect to reduce pulse amplitude in the visible portion of the vein close to the disc center.^8^ It is also known that vessel compliance alterations affect PWVs and that the latter may be a useful marker of vascular disease states altering compliance or stiffness.^4^^,^^9^

Commonly used methods to measure PWV in the body include traditional two point techniques such as the carotid–femoral PWV method, the optical coherence tomography (OCT) two-point method, and continuous approaches that rely on ongoing phase measurements along a vessel. The carotid–femoral PWV method is commonly used for non-invasive arterial stiffness assessment.^4^ The PWV is calculated by measuring the time to trough of an arterial pressure pulse wave at two locations along the carotid and femoral arteries.^10^ The time to trough delay of the arterial pressure pulse wave between the two locations along with the distance between both locations are then used to calculate the carotid–femoral PWV.^10^ Spahr et al.^11^ attempted to measure PWVs along the length of retinal veins using high-speed, phase-sensitive, full-field, swept-source OCT to measure axial expansion of the vessels to determine the time delay of pulsation. They applied the measurement principle to a large artery and vein above the macula in the eye of a healthy subject. However, although they were able to a measure an arterial speed of 620 ± 50 mm/s, they could not detect a time delay for the vein, concluding that no propagation of a single-pressure wavefront can be observed in veins.^11^ We have previously reported a retinal venous PWV of 22.2 mm/s within the central optic disc retrograde to blood flow estimated using manual measures from video recordings.^12^ More recently we reported a median venous PWV of 20.8 mm/s, retrograde to blood flow near the optic disc center in five subjects.^2^ Here, the PWV was that of the first harmonic of the pulse wave, measured using modified photoplethysmography and harmonic regression analysis of image intensity over three cardiac cycles. Very recently, Tang et al.^13^ reported a mean retinal vein PWV in the circumpapillary retinal venous vasculature of 11.1 mm/s in six subjects using a methodology similar to ours but based on the pulsation frequency determined as the frequency with maximum power in the Fourier spectrum.

The retina offers a unique opportunity to non-invasively measure PWVs in the microvasculature because it is the only part of the body where these vessels can be directly seen.^2^^,^^14^ During ophthalmoscopic examination, visible venous wall pulsation is often observed within the veins in the optic disc. The underlying biomechanics of the venous vessel wall pulse wave are complicated and not fully understood.^2^^,^^8^ Venous intraluminal pressure is generally equivalent to the surrounding tissue in the eye.^15^^,^^16^ In the central optic disc region, transmural pressures are close to zero (Fig. 1).^15^^,^^16^ This raises the possibility that pulsation in surrounding tissues will directly affect the vein. Maximum pulsation tends to occur close to the optic disc center and to be in phase with cerebrospinal fluid pressure pulsation. This suggests that a pulse wave propagates retrograde to flow from the retrolaminar location of the optic nerve, where it is surrounded by cerebrospinal fluid.^8^ We do know that the intraocular pressure (IOP) also pulsates and so may also influence retinal vein pulse characteristics. Furthermore in blood vessels there is an association between intravascular pressure and PWV.^17^

**Figure 1.**
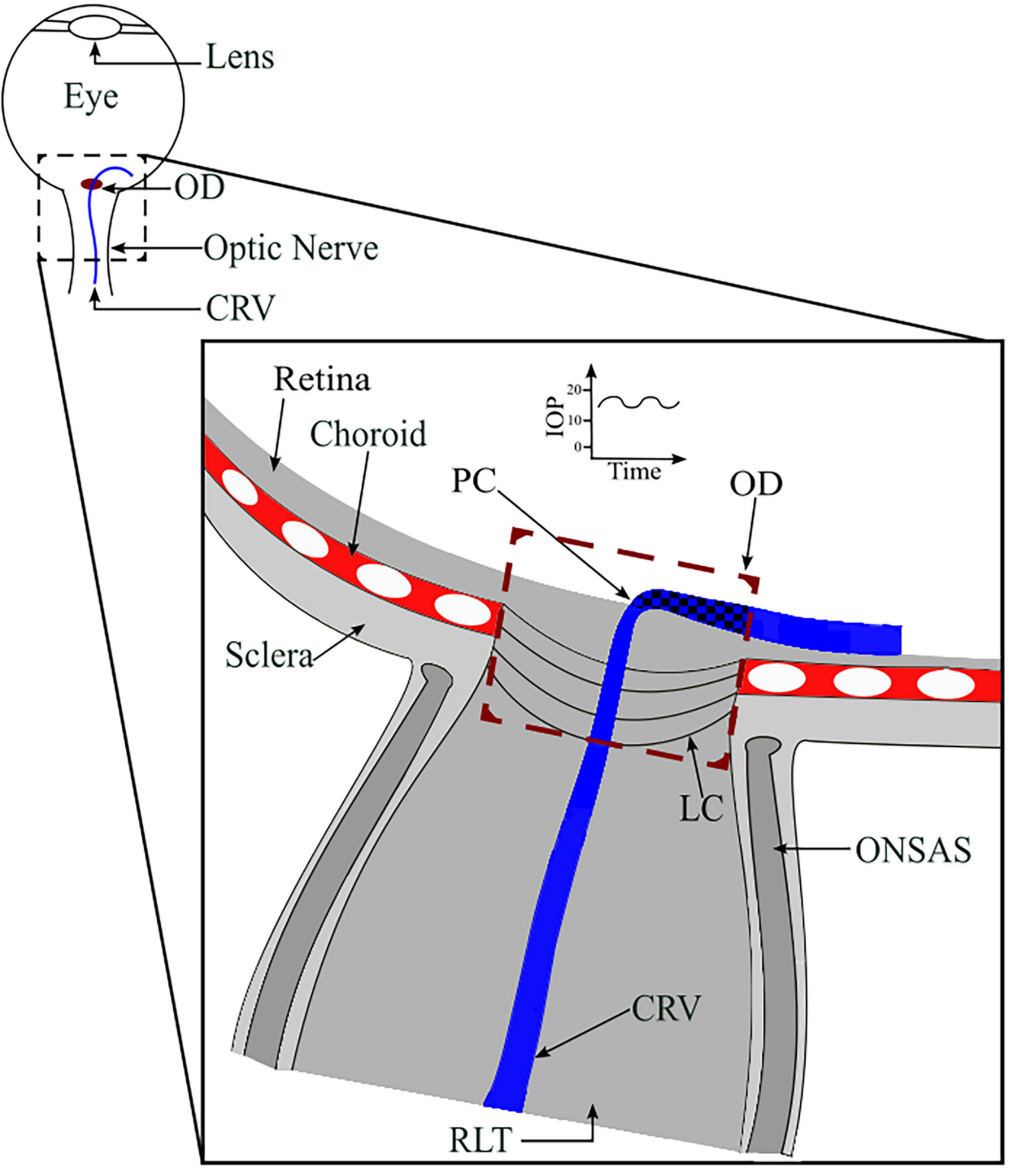
Illustration of a retinal vein traversing the optic disc (OD) from the boundary to the center of the OD (*dashed box*), where it dives down toward the lamina cribrosa (LC) and merges with the central retinal vein (CRV), which is enclosed by the optic nerve subarachnoid space (ONSAS) containing cerebrospinal fluid (CSF). The visible segment of the vein within the optic disc is indicated with a *checkered blue and black pattern*. Partial collapse (PC) of the retinal vein can be seen as it reaches the center of the optic disc due to the drop in transmural pressure. IOP exerts an external pressure on the retinal vein, and, upon exiting the eye posteriorly into the retrolaminar tissue (RLT), the central retinal vein becomes enveloped in cerebrospinal fluid.

To date, no studies have characterized venous pulse wave propagation in the more peripheral aspect of the optic disc. However, in 2021, Bedggood and Metha^18^ estimated PWVs (using adaptive optics imaging) across 74 retinal capillary segments near the macula in three human subjects and reported a mean ± SEM magnitude of 6.4 ± 0.5 mm/s. Interestingly, the velocities were in the direction of flow in 56% of capillaries and opposite to flow in 44%.^18^ In the current study, we applied our recently developed method for measuring PWVs to characterize the propagation distally up to and beyond the point of maximum pulsation in six healthy individuals for varying IOPs.

## Methods

The six subjects (four male, four left eyes) recruited for the study had a mean age of 42.3 ± 18.6 years (range, 26–69). The ages of each subject were as follows: subject A, 26 years old; subject B, 31 years old; subject C, 31 years old; subject D, 34 years old; subject E, 63 years old; and subject F, 69 years old. Participants across a broad age range were selected to reduce the risk of age-related physiological bias. We acquired color videos of the optic nerve head, spanning a minimum of three consecutive cardiac cycles, from six healthy eyes of six individuals over a range of induced IOP (IOPi) values using a modified photoplethysmography technique previously described.^8^^,^^19^^,^^20^ Briefly, for each eye slit lamp (ZEISS, Oberkochen, Germany), video photography using a Canon EOS 5D Mark III (Canon, Tokyo, Japan) was performed with a Meditron ophthalmodynamometer (Meditron, Völklingen, Germany), applying force to the eye in steps. Using baseline IOP and these applied force values, we calculated the IOPi at each step. A video sequence at each step consisting of three cardiac cycle lengths was analyzed. The green color channel frames were extracted, cropped to the size of the optic disc, and spatially aligned to the sharpest frame (affine registration). The optic disc and a selected vein were manually delineated in the sharpest image. The vein mask was used to extract a single-pixel-thick vessel centerline. Starting with the centerline pixel closest to the optic disc center, the sequence of pixels along the centerline was used to define a path. The distance of each pixel along this path from the start was then computed. The distance was calibrated based on the known height of the optic disc in millimeters obtained using a confocal infrared reflectance image captured by the scanning laser ophthalmoscope within a SPECTRALIS OCT system (Heidelberg Engineering, Heidelberg, Germany). The negative logarithm of pixel intensity was multiplied with a hemoglobin absorption factor of 68 (arbitrary units) for each frame so that, based on the Beer–Lambert law, the resulting intensity was proportional to blood column thickness and thus the vessel wall diameter in the axial dimension.^8^^,^^19^^,^^20^ A harmonic regression model, which we have described previously,^12^^,^^21^^,^^22^ was then fitted to the intensity time series at each pixel using generalized least squares, and the change in phase of the first harmonic was used to determine the velocity (i.e., direction and speed) of the pulse wave along the vessel (Supplementary Fig. S1). The model consists of a periodic component, non-periodic component, and an error component. The periodic component captures the pulsatile changes in the signal. It is modeled as a second-order Fourier series in sine–cosine form, where the first harmonic corresponds to the frequency of the cardiac cycle and the second to twice this frequency. It also includes a constant term representing the average value or baseline level of the signal over one cycle. The non-periodic component captures lighting changes between cycles and is modeled using a broken stick model where the intensity trend across cycles is assumed to be piecewise linear. The error component captures the residual variation or noise and is modeled using a first-order autoregressive model (accounts for temporal correlation between frames). From the fitted model, we computed the pulsation amplitude as the difference between the maximum and minimum values of the periodic component. We used the sine and cosine coefficient estimates (means) and standard errors for the first harmonic to estimate a 95% confidence interval (CI) for the phase of the first harmonic. This involved generating 1000 random samples from the normal distributions defined by the means and standard errors of the coefficients, calculating the phase from each sample pair, and calculating the CI from the distribution of phase values.

**Figure 2.**
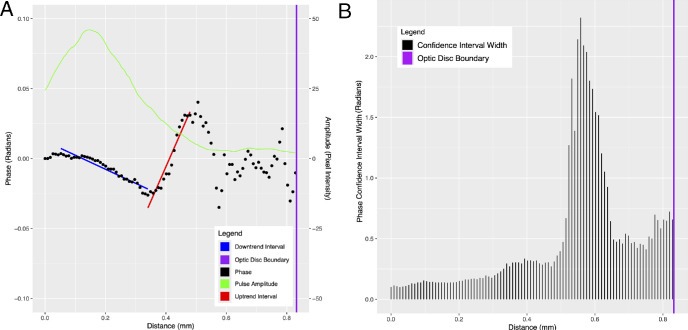
Inferior vein of subject C at an IOPi of 20 mm Hg. (**A**) Plots of phase (*black*) and pulse amplitude (*green*) versus distance along the vessel centerline from the center of the optic disc. The fitted regression lines in the phase down-trend and phase up-trend intervals are shown in *blue* and *red*, respectively. (**B**) Corresponding CI widths for the estimated phase values in **A**.

**Figure 3.**
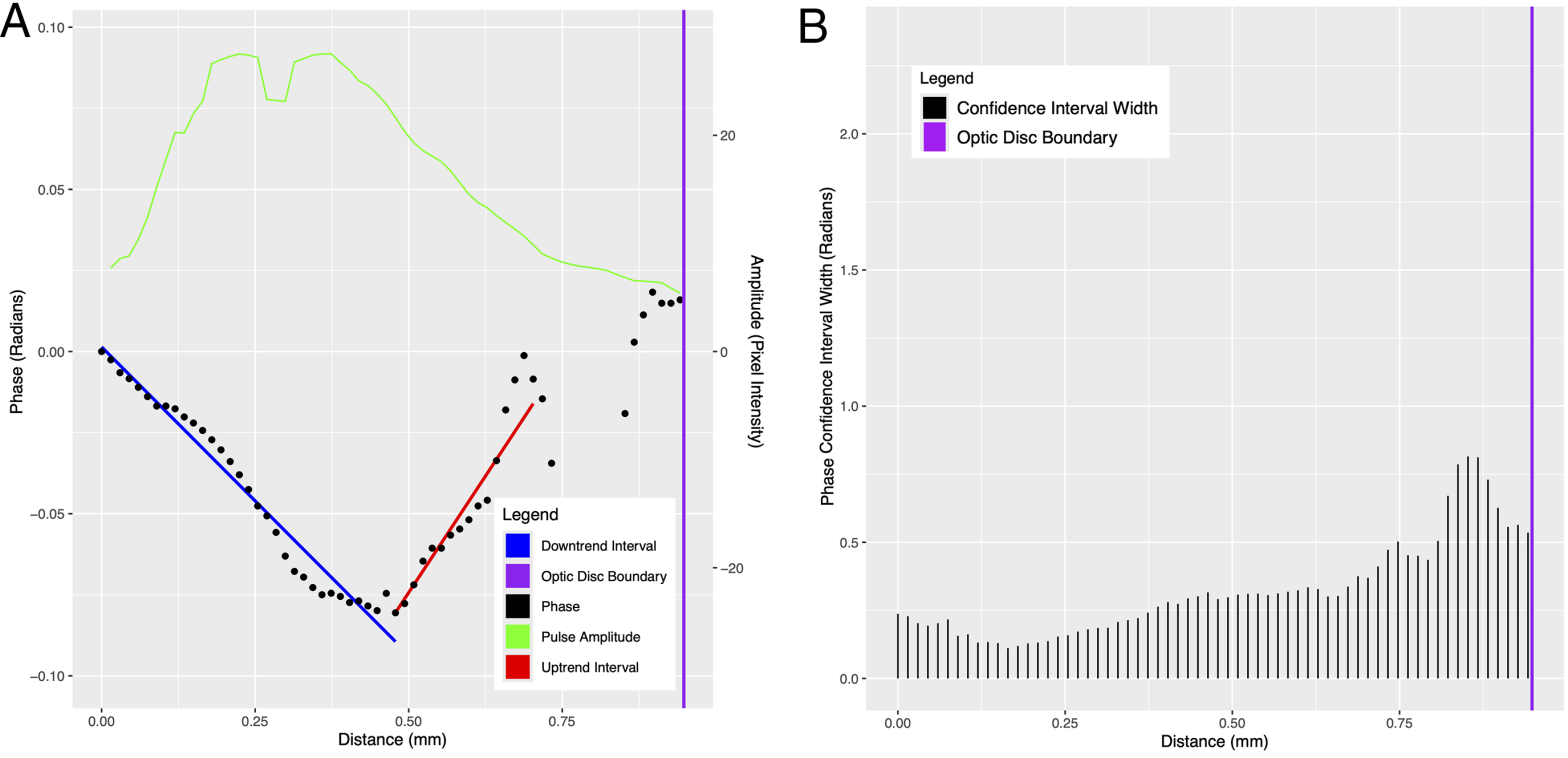
Inferior vein of subject E at an IOPi of 32 mm Hg. (**A**) Plots of phase (black) and pulse amplitude (green) versus distance along the vessel centerline from the center of the optic disc. The fitted regression lines in the phase down-trend and phase up-trend intervals are shown in blue and red, respectively. (**B**) Corresponding CI widths for the estimated phase values in **A**.

The pulsation amplitude and phase (estimate and 95% CI) of the first harmonic were recorded for each vein centerline pixel, and the phase values were normalized so that the first pixel had a value of zero (by subtracting the initial value from all values). The normalized phase (and 95% CI) and the pulsation amplitude were both plotted against centerline distance. In all cases, the plots showed an initial linear down trend followed by a linear up trend (Figs. 2, 3). We estimated the PWV in the initial linear down trend using the method we previously described.^2^ Briefly, a least-squares line was fitted to the distance–phase points in the closed interval defined by the first local maximum closest to the origin, and the first local minimum was referred to as the phase turning point. The slope of this line is phase (radians) per unit distance (mm). After converting the phase to seconds (based on the average cardiac cycle time), the inverse of the slope is the PWV (mm/s). We similarly estimated the PWV in the linear up trend by using the least squares fit to the interval defined by the turning point (end of the down trend) and the subsequent local maximum. The interval was chosen because in all cases the subsequent phase trajectory showed no clear trend, and phase CIs were typically much larger (Figs. 2, 3). The pracma package in R (R Foundation for Statistical Computing, Vienna, Austria)^23^ was used to find the first significant local maximum past the turning point.^24^

For each video, the turning point distances from the position of maximum pulse amplitude and optic disc center were measured. The distances were measured along the vessel centerline path, and in the latter case additionally included the Euclidean distance from the optic disc center to the start of the path. The distances were measured in both millimeter and scalar multiples of what we refer to as the hump width. The hump width is a measure of the width of the maximum pulse amplitude peak, which varies by video (i.e., by subject and pressure). It is defined to be twice the standard deviation of the Gaussian curve fitted to the peak in the distance–pulse amplitude curve corresponding the maximum pulse amplitude. The nls package in R was used to perform the nonlinear least-squares curve fitting.^25^^,^^26^ Finally, the distance of the position of maximum pulse amplitude from the optic disc center (distance defined in the same way as for the turning point) was measured in millimeters.

The use of human subjects for the photoplethysmography measurements was approved by the Belberry Human Research Ethics Committee (permit no. 2015-11-756-A-2), in accordance with the tenets of the Declaration of Helsinki and in compliance with National Health and Medical Research Council guidelines for clinical trials. All measurements were performed according to relevant guidelines and regulations, and informed consent was obtained from all participants.

### Statistical Analysis

All statistical analyses were performed in R using the α = 0.05 level of significance. The package nlme^27^ was used for fitting linear mixed models. Backwards elimination was used to iteratively remove uninformative explanatory variables in each linear mixed model; at each step the variable with the highest *P* value was identified and removed if its *P* value was greater than the level of significance. Diagnostic plots were used to assess whether fitted linear mixed models satisfied the two key model assumptions: normality of the residuals and homogeneity of variance. Normality of residuals was assessed using a *Q*–*Q* plot. Homogeneity of variance was assessed using a plot residuals versus fitted values. Where necessary, the response variable was transformed to satisfy these assumptions. The PairedData package in R was used to perform the Pitman–Morgan test of equality of variances for paired samples.^28^

#### Investigating the Certainty of Phase Estimates Before and After the End of the Linear Up Trend

The CI widths of the estimated phase values before and after the end of the linear up trend were summarized across all pressures for each subject using side-by-side box plots. The CI widths for each subject and pressure were normalized by dividing by the smallest interval width. A linear mixed model was then fitted to the data from all videos to investigate whether there was a difference in normalized phase CI widths before and after the end of the linear up trend. The explanatory variables (fixed effects) were position (either before or after the end of the linear up trend) and age and sex. The subject identifier was included in the model as a random effect (allowing the model to account for individual difference between subjects and for repeated measurements within the same subject).

#### Comparison of Turning Point Distances From the Maximum Pulse Amplitude and Optic Disc Center Over all Subjects for a Matched Range of Induced IOPs

Five videos were selected for each subject, similarly matched in terms of IOPi values, with pressures ranging from 16 to 45 mm Hg. This was done to ensure that all subjects were equally represented in the subset of videos. The turning point distances (both in millimeters and scalar multiples of hump width) from the maximum pulse amplitude location and optic disc center were summarized numerically in terms of minimums, maximums, means, standard deviations, medians, and interquartile ranges (IQRs). They were also summarized graphically using box plots. Finally, the distances were averaged over the five pressures for each subject, and Pitman–Morgan tests were performed to test for differences in the variances of turning point distances from maximum pulse amplitude and from optic disc center.

#### Investigating Which Variables Were Predictive of the Locations of the Turning Point and Maximum Pulse Amplitude

Five linear mixed models were fitted to the data from all videos to investigate which variables were predictive of the location of the turning point and separately the maximum pulse amplitude. The five response variables used were (1) D1, which is the distance of the turning point from the position of maximum pulse amplitude in millimeters; (2) D2, which is the distance of the turning point from the center of optic disc in millimeters; (3) D3, which is D1 measured in terms of hump width; (4) D4, which is D2 measured in terms of hump width; and (5) D5, which is the distance of the location of maximum pulse amplitude from the center of the optic disc in millimeters. The explanatory variables (fixed effects) for the first four models were IOPi, pulse wave speed (PWV magnitude) before the turning point (S1), pulse wave speed after the turning point (S2), mean pulse amplitude, age, and sex. The models also included interaction between IOPi and S1 and between IOPi and S2. The fifth model included the same explanatory variables and additionally D2. Interactions between pressure and speed were included given the likely relationship between wave velocity and pressure.^17^ The subject identifier was included in each model as a random effect.

#### Investigating Which Variables Were Predictive of PWV

PWVs before and after the turning point were summarized across all pressures for each subject in terms of medians and interquartile ranges and graphically using box plots. Four linear mixed models were then fitted to the data from all videos to investigate which variables were predictive of the pulse wave speed before the turning point (S1) and after the turning point (S2). The response variable for the first model was S1 and the explanatory variables were IOPi, S2, mean pulse amplitude, age, and sex. The response variable for the second model was S2, and the explanatory variables were the same as for the first model except that S2 was replaced with S1. The models also included interaction between IOPi and S1 and between IOPi and S2, respectively. The third and fourth models were the same as the first and second models, respectively, except that the explanatory variable mean pulse amplitude was replaced with maximum pulse amplitude (highly collinear). The subject identifier was included in each model as a random effect. The intraclass correlation coefficient for each response variable was calculated by dividing the random effect variance by the intercept-only model variance.

## Results

Optic nerve head videos were acquired for each eye for IOPi values ranging from 12 to 61 mm Hg with a mean IOP of 33.2 ± 13.3 mm Hg. A total of 57 videos were acquired.

**Figure 4.**
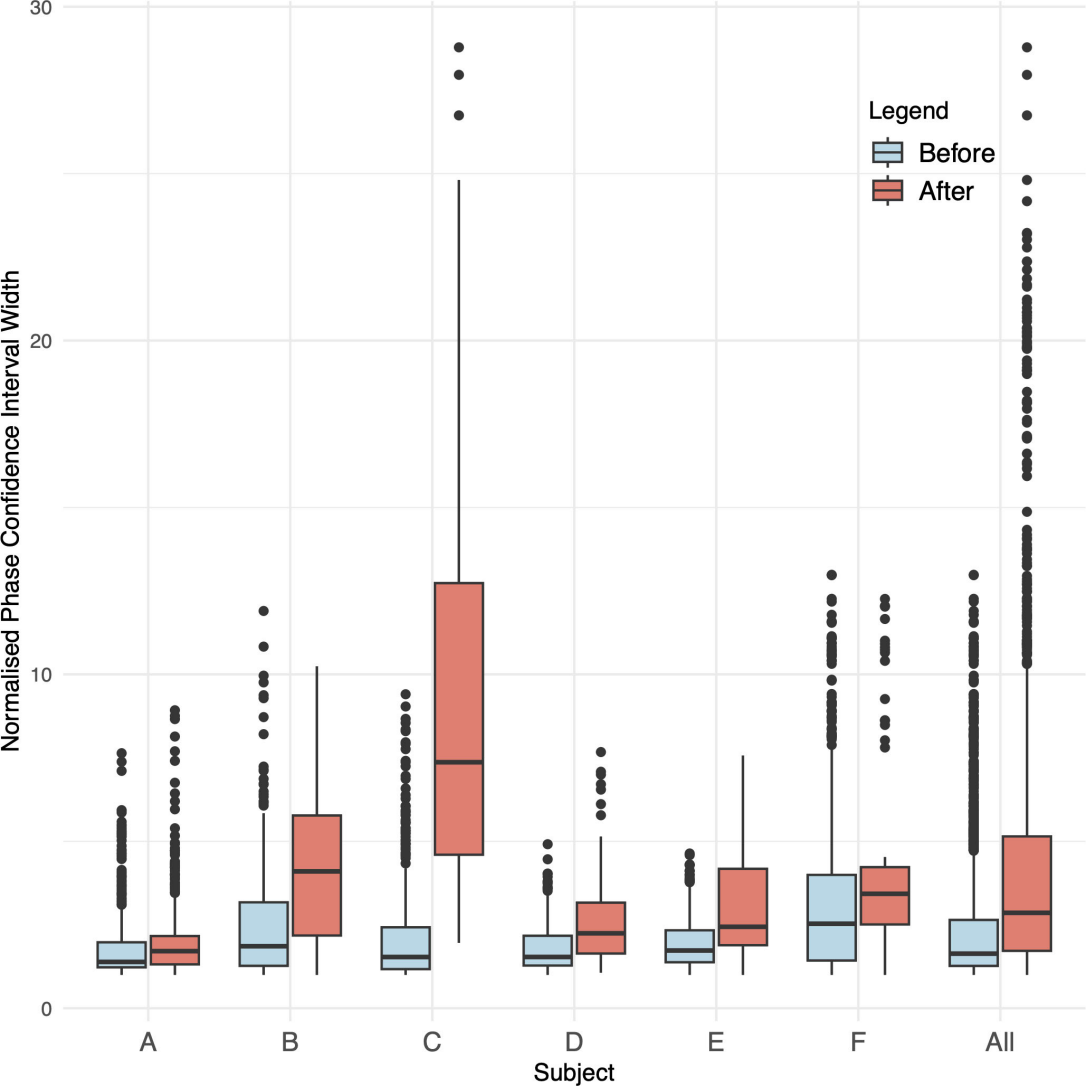
Normalized CI widths of the phase measurements before and after the end of the linear up-trend interval.

Figures 2A and 3A show plots of phase (black) and pulse amplitude (green) versus distance along the vessel centerline for two representative cases (both inferior veins). There is no evidence of phase wrapping. The fitted regression lines to the distance–phase points in the down-trend interval before the turning point (blue line) and the up-trend interval after the turning point (red line) are also shown. Figures 2B and 3B show plots of the CI widths for the phase estimates in Figures 2A and 3A, respectively. Figures 2 and 3 show that, beyond the up-trend interval to the optic disc boundary (purple line), the phase estimates have the largest uncertainty (largest CIs), and the phase trajectory has no discernible trend. These observations hold true in general for the remaining cases (plots not shown). This can be seen in Figure 4, which shows side-by-side box plots of the CI widths of the estimated phase values before and after the end of the up-trend interval for each subject. The difference before and after was assessed using the fitted linear mixed model and found to be statistically significant (*P* < 0.001, *c* = −0.13).

**Figure 5.**
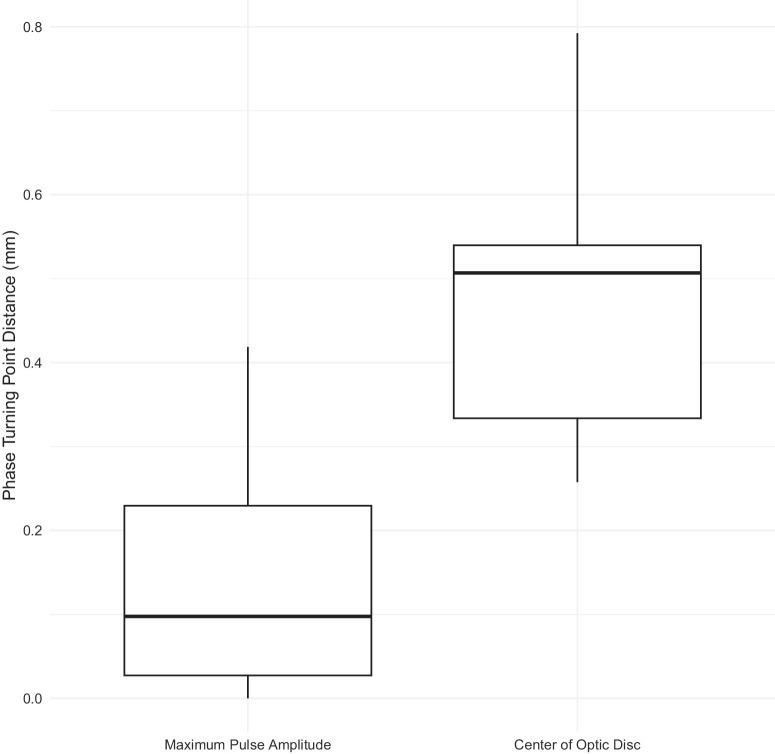
Box plots of the distances of the phase turning point from the position of maximum pulse amplitude and the center of the optic disc (in millimeters) over all subjects for a matched range of IOPi values.

**Figure 6.**
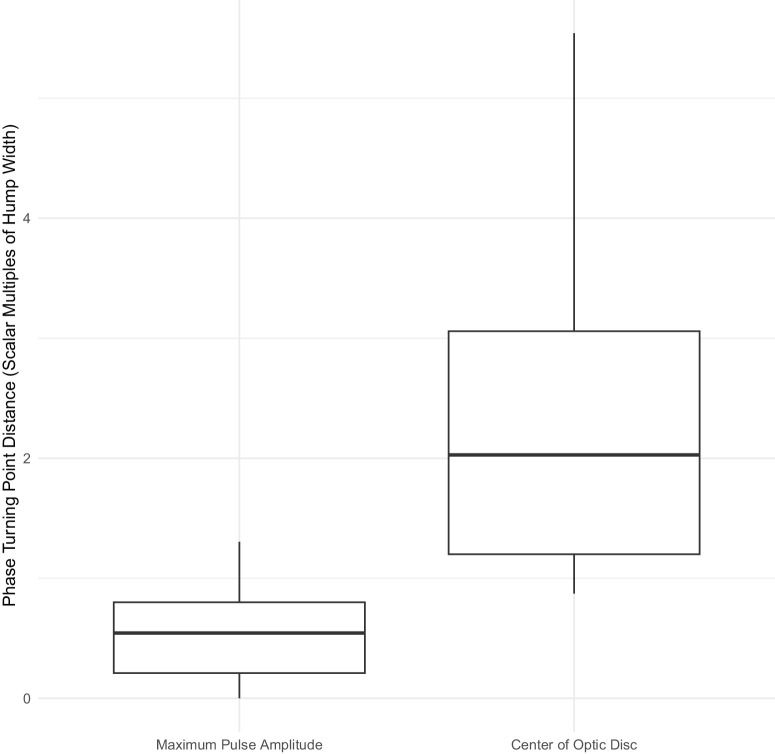
Box plots of the distances of the phase turning point from the position of maximum pulse amplitude and the center of the optic disc (in scalar multiples of the hump width) over all subjects for a matched range of IOPi values.

The phase turning point distances from the maximum pulse amplitude and optic disc center for the five videos selected for each subject (matched range of IOPi values) are summarized in Table 1 and as box plots in Figures 5 and 6. In all cases, the turning point was located just distal to the point of maximum pulse amplitude for both distance units (millimeters and scalar multiples of hump width). A statistically significant difference in the variances of the turning point distances from the maximum pulse amplitude and from the optic disc center was found for the hump width–based units (Pitman–Morgan test, *P* = 0.019) but not for millimeters. This suggests that the distances from the maximum pulse amplitude are less variable than those from the optic disc center when measured in terms of hump width. The median of the median hump widths across all pressures for each subject (Supplementary Table S1) was 0.28 mm (IQR = 0.22).

**Table 1.** Summary of Phase Turning Point Distances Over All Subjects for a Matched Range of Induced IOPs

Phase Turning Point Distance	Minimum	Maximum	Mean	SD	Median	IQR
D1 (mm)	0	0.42	0.14	0.12	0.10	0.20
D2 (mm)	0.26	0.79	0.46	0.14	0.51	0.21
D3 (scalar multiples of HW)	0	1.30	0.49	0.34	0.54	0.59
D4 (scalar multiples of HW)	0.87	5.54	2.34	1.28	2.03	1.86

D1 is the distance from the location of maximum pulse amplitude in mm, D2 is the distance from the centre of optic disc in mm, D3 is D1 but measured in terms of hump width and D4 is D2 but measured in terms of hump width.

Table 2 summarizes the five linear mixed models fitted to the data from all videos to investigate which variables were predictive of the location of the turning point and separately the maximum pulse amplitude. We found statistically significant relationships between the distance of the turning point from the position of maximum pulse amplitude in millimeters (D1) and each of S1, IOPi, S2, age, and the interaction of IOPi and S2. However, we found a statistically significant relationship only between the same distance measured in terms of hump width (D3) and the interaction of IOPi and S2. We also found a statistically significant relationship between the distance of the turning point from the center of the optic disc measured in terms of hump width (D4) and age. Finally, we found statistically significant relationships between the distance of the location of maximum pulse amplitude from the center of the optic disc in millimeters and each of S2, D2, and the interaction of IOPi and S2.

**Table 2.** Summary of the Five Linear Mixed Models Fitted to the Data From All Videos Used to Predict Phase Turning Point Locations and Maximum Pulse Amplitudes

		Explanatory Variable *P* Values (Coefficient)					
Response Variable	Unit	S1	IOPi	S2	Age	IOPi: S2^*^	D2^†^
(D1)^0.5^	mm	0.023 (0.0024)	0.018 (0.0050)	0.045 (0.0058)	0.016 (0.0071)	0.0017 (−0.00026)	NA
D2	mm	—	—	—	—	—	NA
(D3)^0.6^	HW	—	—	—	—	0.0054 (−0.00055)	NA
(D4)^−0.1^	HW	—	—	—	0.019 (0.0028)	—	NA
(D5)^0.3^	mm	—	—	0.0004 (−0.0044)	—	0.0001 (0.00014)	0.013 (0.28)

D1 is the distance of the turning point from the position of maximum pulse amplitude in mm, D2 is the distance of the turning point from the center of optic disc in mm, D3 is D1 measured in terms of hump width, D4 is D2 measured in terms of hump width and D5 is the distance of the location of maximum pulse amplitude from the center of the optic disc in mm.

* Interaction between two predictors.

† Explanatory variable specific to response variable D5.

**Figure 7.**
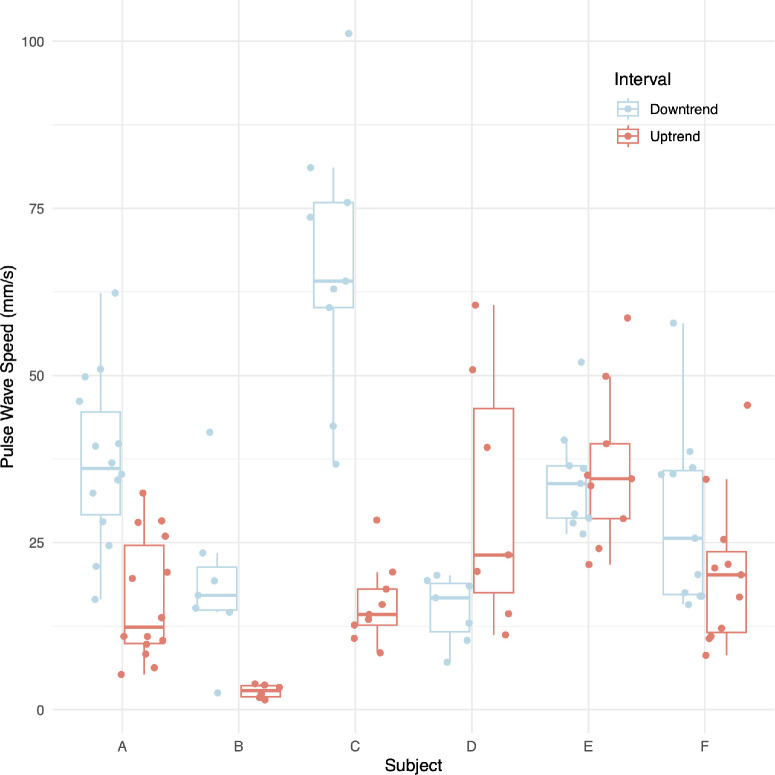
Comparison of pulse wave speeds (PWV magnitudes) between the down-trend and up-trend intervals across all pressures for each subject.

For all videos, the PWVs in the down-trend interval were negative, being retrograde to flow, with the regression line having a negative slope. Likewise, PWVs in the up-trend interval were positive, being orthograde to flow with the regression line having a positive slope. The PWVs before and after the turning point across all pressures for each subject are summarized numerically in Table 3 and graphically in Figure 7. Based on the four linear mixed models fitted to the data from all videos, we found statistically significant relationships between pulse wave speed in the down-trend interval, (S1)^0.^5, and both mean pulse amplitude (*P* = 0.0013, *c* = 0.16) and maximum pulse amplitude (*P* < 0.001, *c* = 0.072). We also found statistically significant relationships between pulse wave speed in the up-trend interval, (S2)^0.^1, and both mean pulse amplitude (*P* = 0.017, *c* = 0.0081) and maximum pulse amplitude (*P* = 0.032, *c* = 0.0031). There were no statistically significant relationships between either of the pulse wave speeds and any of the other explanatory variables including IOPi. The intraclass coefficients of S1 and S2 were 0.66 and 0.51, respectively.

**Table 3.** Summary of PWV Results for all Subjects

	Down-Trend Interval		Up-Trend Interval	
Subject	Median (mm/s)	IQR	Median (mm/s)	IQR
A	−36.07	15.36	12.37	14.67
B	−18.07	5.27	2.84	1.64
C	−64.12	15.73	14.24	5.38
D	−14.84	8.78	21.91	23.09
E	−33.84	7.84	34.55	11.20
F	−25.65	18.48	20.18	12.04
Mean	−32.40	22.18	19.63	17.58

## Discussion

We confirmed that venous PWV estimated from the first harmonic is retrograde to flow in the initial central disc segment of vein up to and generally just beyond the point of maximum pulsation. More distal to this region it changes direction, moving toward the optic disc center. The more central retrograde wave has been described in our earlier publication and probably results from the optic nerve subarachnoid space pulsation acting as a pulse wave generator, sending a wave towards the eye and onto the central disc segment of vein.^2^

**Figure 8.**
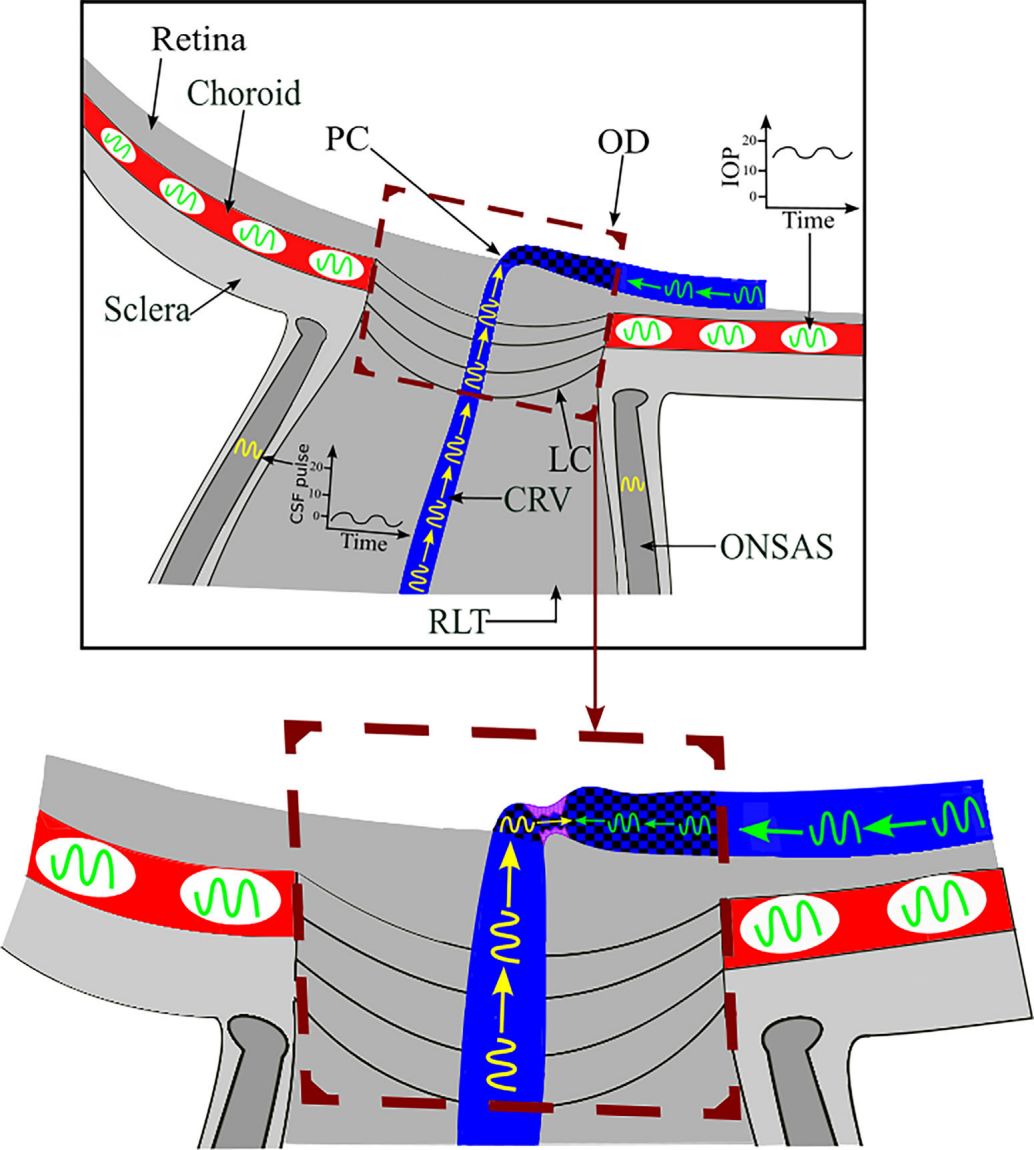
Illustration showing how two pulse wave generators may give rise to opposing pulse waves in a retinal vein segment (*checkered blue and black pattern*) within the optic disc (OD) (see also the caption for Fig. 1). The pulse wave generated from the optic nerve subarachnoid space (ONSAS) is transmitted to the retinal vein and travels in the opposite direction to blood flow (*yellow arrows*). The pulse wave generated from the vitreous chamber (IOP) is transmitted to the retinal vein via the choroid and travels in the direction of blood flow (*green arrows*). The retinal vein exhibits partial collapse (*purple shading*) at a location distal to the OD center, occurring just proximal to the point where the two opposing waves meet.

The more distal venous segment appears to transmit a pulse wave coming from the opposite direction with a velocity orthograde to blood flow. However, the etiology of this opposing pulse wave is unknown. We do consider the possibility that a second pulse generator may exist, exerting an intermittent force upon the more distal vein within the retina and optic disc boundary. It is known that the choroidal volume expands and contracts during the cardiac cycle and is the main driver of IOP pulsation.^29^ This IOP and choroidal pulsation may intermittently compress the retinal vein on the retinal surface, acting as a second pulse wave generator. Although still speculative, this may induce a pulse wave to travel along the vein from the optic disc boundary toward optic disc center (Fig. 8). The interaction of the two opposing waves may be influenced by the fact that they are in similar phase with identical frequency, due to the constancy of the cardiac cycle.^30^ We can find no mathematical models of two opposing waves traveling along an elastic vessel in the literature. However, a previous study by Bedggood and Metha^18^ also found opposing pulse waves in retinal vessels. They reported that, in 44% of retinal capillaries, pulse waves traveled in the opposite direction to blood flow. They concluded that these results were most likely wave reflections from later branch points due to impedance mismatches.^18^ However, our current findings suggest the possibility that the retrograde pulse waves observed in capillaries could be extensions of retrograde pulse waves found close to the optic disc center. Furthermore, the downstream effects of arterial pulsation may also potentially act as a third pulse generator. A previous study has shown, however, that the arterial pressure pulse does not significantly transmit through the capillary circulation to the venous circulation directly.^31^ We cannot find work examining the propagation of vessel wall pulse from the arterial circulation through the capillary to the venous circulation. With this lack of evidence, this third pulse wave generator remains a possibility, although the lack of significant pressure pulse transmission would appear to reduce the likelihood.

In the absence of a mathematical model to describe this phenomenon we propose that the interaction may result in a focal increase in pulse amplitude near the meeting point of the two waves. We refer to this point as the turning point because mathematically this is where the change in direction occurred. There are some physical analogies to the cardiac cycle generating postulated cerebrospinal fluid pressure pulse and IOP pulse venous waves at equivalent frequencies but in opposite directions. An example could be when a wave approaches an island close to shore forcing two waves to form on the lee side of the island traveling in opposite directions and resulting in a summative wave when they meet. The close relationship of the turning point to the point of maximum pulsation is supported by the observation that it was only a mean of 0.14 mm distal to the maximum pulse amplitude. We found a statistically significant relationship between the distance of the turning point from the maximum pulse amplitude and the interaction between IOPi and the pulse wave speed in the up-trend interval (S2). Specifically, as the product of IOPi and S2 increases, the distance between the turning point and point of maximum pulse amplitude decreases, indicating that S2 becomes more dominant at higher IOPi. We speculate that the observed shift in the location of the turning point is explained by the nature of wave superposition.^32^ When the speed of the antegrade pulse wave and IOPi increase, the antegrade pulse wave becomes more dominant within the composite waveform. Consequently, the turning point in the waveform shifts in the direction of the antegrade propagation (i.e., toward the optic disc center), reflecting the greater influence of the antegrade wave along the retinal vein.

We did not find any evidence of a relationship between the speeds of the pulse waves (S1 and S2) either side of the turning point and IOPi. However, Bramwell and Hill^17^ found in an isolated human carotid artery that the PWV rapidly increased as intravascular pressure was elevated above 60 mm Hg. They also measured two PWVs below 60 mm Hg, at 25 mm Hg and 57 mm Hg, and found the reverse to be true—a slightly faster PWV at the lower pressure.^17^ It is possible that no association was found between pulse wave speed and IOPi in our study because the IOPi values were too low (range, 12–61 mm Hg) for any statistically significant association to be detected. We found that, as the speeds S1 and S2 increased, so too did the mean pulse amplitude and maximum pulse amplitude. The intraclass correlation coefficients for both S1 and S2 were 0.66 and 0.51, respectively, demonstrating a moderately consistent wave speed within individuals across varying IOPi.

Previous studies exploring pulse wave propagation in retinal veins have also noted a peculiar wave behavior in retinal veins. Spahr et al.^11^ utilized a two-point method and hypothesized that multiple waves travel along the retinal vein and interfere with varying time delays, and as such the propagation of a single pressure wavefront is not observable. However, our methodology allows the measurement of phase at each pixel along the retinal vein and analysis of the resultant pulse wave as it propagates along the retinal vein. If one were to take two points along the retinal vein from our distance–phase plots shown in Figures 2A and 3A, the slopes and therefore velocity calculated would vary greatly depending on which two points were selected. The phase analysis at each pixel allows us to track the dominant wavefront as it propagates along the retinal vein within the optic disc boundary.

A canine study in the ascending aorta found that lower order harmonics, including the first harmonic, overestimated the true PWV as measured by the wavefront velocity.^33^ This contrasts with an earlier canine study that found that the first harmonic had a relatively good agreement with the wavefront velocity when compared to the higher order harmonics.^34^ In both studies, the wavefront velocity was measured by placing two arterial catheters spaced 5.0 cm apart in the aorta of a dog, and the time delay between the foot of each pressure wave was then used to calculate wavefront velocity.^33^^,^^34^ In both cases, it was suggested that the PWVs at lower order harmonics are influenced by reflected waves.^33^^,^^34^ The earlier study provides evidence that reflections from arterial branches have the largest influence.^34^ Given that we measured PWVs in small retinal vein segments (<1 mm) with no branching, the effect and existence of reflected waves on the first harmonic PWV in retinal veins would most likely be smaller compared to that in the macrovasculature. However, we do acknowledge the possibility that the use of the first harmonic may overestimate the true PWV.

We found a statistically significant difference in the certainties (CI widths) of phase estimates before and after the end of the phase up-trend interval. The estimates after were larger, and the phase trajectory had no discernible trend. Consequently, we cannot comment on the direction of pulse wave propagation in more distal sections of the vein. This is a limitation of our approach. Individualized ocular biometric data were not used to calibrate the SPECTRALIS OCT system, which may have introduced lateral scaling errors into our optic disc height measurements. A previous study found a 3.0% relative mean difference between scans with and without individualized ocular biometric data.^35^ However, although such errors might in turn affect the magnitudes of the velocity measurements, they would not change the directions. Another limitation of our study is that, although 57 videos were analyzed (acquired over a range of pressures), they were obtained from only six subjects. We did not have any convergence issues when fitting our linear mixed models, but we acknowledge that this small sample size limited the statistical power of our tests.

Collectively, our results suggest that two opposing pulse waves propagate along retinal vein segments within the optic disc. To the best of our knowledge, this is the first time opposing waves have been observed within the same retinal vein close to the optic disc center. The speeds of the two waves and their meeting point appear to influence both the size and location of maximum pulsation amplitudes.

## Supplementary Material

Supplement 1
