# Supplementary material for: Evidence for the Existence of Two Opposing Pulse Waves in Retinal Vein Segments Within the Optic Disc
Source: Invest Ophthalmol Vis Sci. 2025 Dec 15;66(15):43. doi: 10.1167/iovs.66.15.43 (PMC12710786; doi:10.1167/iovs.66.15.43)
Supplement: Supplement 1 [file iovs-66-15-43_s001.docx]

Supplementary Material

Table S1: Summary of 1 HW to mm for each subject

| Subject | Min (mm) | Max (mm) | Mean (mm) | Median (mm) | IQR (mm) | Sd (mm) |
| --- | --- | --- | --- | --- | --- | --- |
| A | 0.15 | 0.21 | 0.17 | 0.17 | 0.03 | 0.02 |
| B | 0.06 | 0.17 | 0.12 | 0.12 | 0.06 | 0.05 |
| C | 0.25 | 0.34 | 0.30 | 0.29 | 0.04 | 0.03 |
| D | 0.07 | 0.15 | 0.11 | 0.12 | 0.04 | 0.03 |
| E | 0.28 | 0.43 | 0.37 | 0.37 | 0.00 | 0.05 |
| F | 0.33 | 0.53 | 0.39 | 0.38 | 0.00 | 0.05 |
| All | 0.06 | 0.53 | 0.26 | 0.28 | 0.22 | 0.12 |


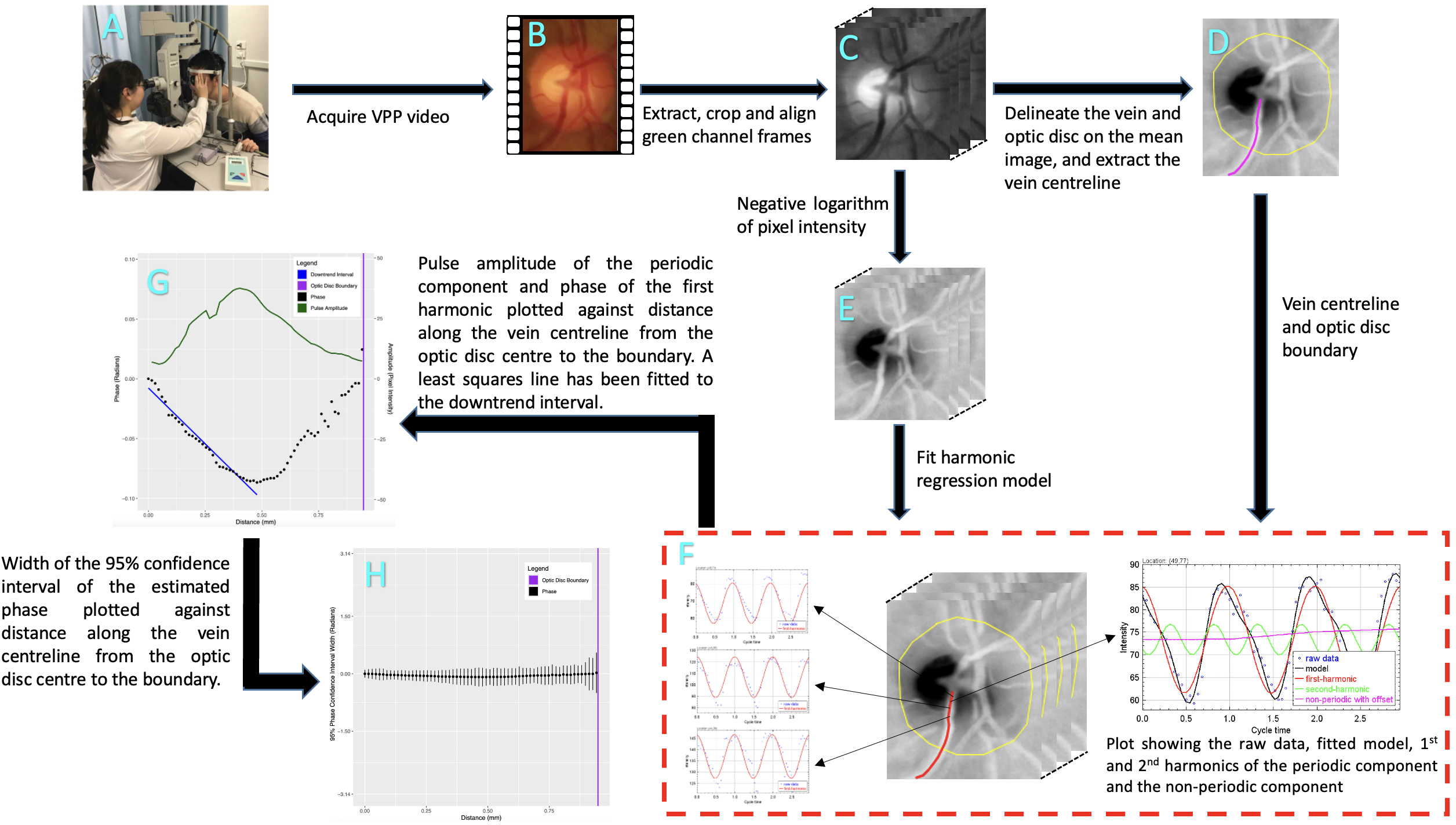


*Supplementary figure 1:* Method for calculating pulse wave velocity. (A) Patient is seated at the video ophthalmodynamometer with the pulse oximeter attached to their left index finger. (B) Acquired video frames over 3 cardiac cycles. (C) Extracted green channel frames (shown in greyscale) (D) Manual delineation of the optic disc boundary and the retinal vein. The centreline is given by the skeleton (medial axis) of the vessel mask. (E) Negative log transform of the pixel intensities in *(C)* so that intensity is related to blood column thickness and hence vessel diameter (Beer-Lambert law). (F) The harmonic regression model is fitted to each pixel time series in *(E)*. The fitted model, first harmonic component, second harmonic component, and non-periodic component are shown for one of the centreline pixels. The first harmonic of the periodic component is shown for 3 centreline pixels. (G) Pulse amplitude of the periodic component and phase of the first harmonic plotted against distance along the vein centreline from the optic disc centre to the boundary. The phase trajectory is used to compute the velocity (i.e., speed and direction) of the pulse wave. For example the velocity in the downtrend interval is computed from the inverse of the slope of the least squares line fitted to this interval. (H) Width of the 95% confidence interval of the estimated phase in (*G)* plotted against distance along the vein centreline from the optic disc centre to the boundary.
